# Elderly and Nonelderly Use of a Dedicated Ambulance Corps' Emergency Medical Services in Taiwan

**DOI:** 10.1155/2016/1506436

**Published:** 2016-07-11

**Authors:** Chien-Chia Huang, Wei-Lung Chen, Chien-Chin Hsu, Hung-Jung Lin, Shih-Bin Su, How-Ran Guo, Chien-Cheng Huang, Pi-Ching Chen

**Affiliations:** ^1^Tainan City Government Fire Bureau, Annan Branch, Tainan 709, Taiwan; ^2^Chang Jung Christian University, Tainan 711, Taiwan; ^3^Department of Emergency Medicine, Cathay General Hospital, Taipei 106, Taiwan; ^4^School of Medicine, Fu Jen Catholic University, Taipei 242, Taiwan; ^5^Department of Emergency Medicine, Chi-Mei Medical Center, Tainan 710, Taiwan; ^6^Department of Biotechnology, Southern Taiwan University of Science and Technology, Tainan 710, Taiwan; ^7^Department of Emergency Medicine, Taipei Medical University, Taipei 110, Taiwan; ^8^Department of Occupational Medicine, Chi-Mei Medical Center, Tainan 710, Taiwan; ^9^Department of Leisure, Recreation and Tourism Management, Southern Taiwan University of Science and Technology, Tainan 710, Taiwan; ^10^Department of Medical Research, Chi-Mei Medical Center, Liouying, Tainan 736, Taiwan; ^11^Department of Environmental and Occupational Health, College of Medicine, National Cheng Kung University, Tainan 701, Taiwan; ^12^Department of Occupational and Environmental Medicine, National Cheng Kung University Hospital, Tainan 701, Taiwan; ^13^Bachelor Program of Senior Service, Southern Taiwan University of Science and Technology, Tainan 710, Taiwan; ^14^Department of Geriatrics and Gerontology, Chi-Mei Medical Center, Tainan 710, Taiwan

## Abstract

*Backgrounds and Aim*. Taiwan's population is gradually aging; however, there are no comparative data on emergency medical services (EMS) use between the elderly and nonelderly.* Methods*. We analyzed the emergency calls dealt with between January 1 and April 4, 2014, by EMS in one city in Taiwan. All calls were divided into two groups: elderly (≥65 years) and nonelderly (<65 years). Nontransport and transport calls were compared between the groups for demographic characteristics, transport time, reasons for calling EMS, vital signs, and emergency management.* Results*. There were 1,001 EMS calls: 226 nontransport and 775 transport calls. The elderly accounted for significantly (*P* < 0.05) fewer (28 (9.2%)) nontransport calls than did the nonelderly (136 (21.4%)). In the transport calls, 276 (35.6%) were the elderly. The elderly had a higher proportion of histories for cardiovascular disease, cerebrovascular disease, hypertension, diabetes, end-stage renal disease, cancer, Parkinson's disease, and Alzheimer's disease. In addition, the elderly had significantly longer total transport time, more nontrauma reasons, and poorer consciousness levels and lower oxygen saturation and needed more respiratory management and more frequent resuscitation during transport than did the nonelderly.* Conclusion*. The elderly have more specific needs than do the nonelderly. Adapting EMS training, operations, and government policies to aging societies is mandatory and should begin now.

## 1. Introduction

Rapid aging of the general population is occurring all over the world, especially in developed countries [1, 2]. Taiwan became the “aging society” (≥65 years = 7%) in 1994 and the “aged society” (≥65 years = 14%) estimated in 2018, and it is estimated to become the “super-aged society” (≥65 years = 20%) in 2025 [1]. The aging rate in Taiwan is three times that of the US and it is one of the most rapidly aging countries in the world [1, 2]. Aging has become one of the most important issues in Taiwan [1].

Many studies [3, 4] report that the need for emergency medical services (EMS) is growing because of the rapidly aging population. A study in a US emergency department (ED) reported that the proportion of patients using EMS to reach the ED increases steadily with age [3]. It estimated that the elderly will account for approximately half of EMS transports by 2030. This trend is international and highlights the growth of EMS needs for the elderly and the importance of emphasizing elderly care in EMS training. In a Canadian study [4], the elderly were responsible for half of the EMS calls in 2010. To the best of our knowledge, however, there has been no study comparing EMS use between the elderly and nonelderly in Taiwan in the past two decades. Therefore, we did this pilot study in dedicated ambulance corps to clarify whether the elderly need more EMS resources because of their frailer health. We expected that the result could help us improve the prehospital care and modify the standard operation procedure and government policies for the elderly in the future.

## 2. Materials and Methods

### 2.1. Study Design, Setting, and Participants

**Figure 1:**
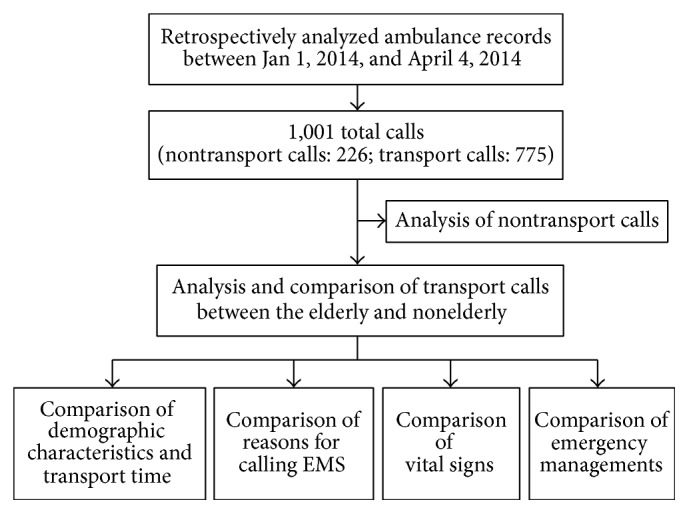
Flowchart of this study. EMS, emergency medical services.

This retrospective study used data from a dedicated ambulance corps in Tainan City, Taiwan, between January 1 and April 4, 2014. In 2014, Tainan's population was 1.9 million with no significant difference in males and females and about 240,000 (12.62%) elderly (≥65 years) residents [5]. All of the ambulance records during the study period—patient age, sex, reasons for calling EMS, vital signs, emergency management, the number of nontransport (the EMS was called, but the patient was not taken to the hospital by EMS ambulance) and transport (the EMS was called, and the patient was taken to the hospital by EMS ambulance) calls, and transport time—except for those with incomplete data, were analyzed (Figure 1). The criteria and reasons of nontransport calls are the follows: (1) the person involved refusing transport; (2) police officer taking over the task; (3) mission canceled on the way; (4) misstatement; and (5) others.

### 2.2. Comparison between the Elderly and Nonelderly

We first compared nontransport versus transport calls between the elderly and nonelderly. We then compared demographic characteristics, transport time, reasons for calling the EMS, and emergency management between the elderly and nonelderly in the transport calls (Figure 1).

### 2.3. Ethics Statements

This study was done in accord with the Declaration of Helsinki and was approved by the Institutional Review Board (IRB) at Chi-Mei Medical Center (IRB number 10408-010). Because this was a retrospective data review, patient informed consent was waived. Neither patient human rights nor welfare was affected.

### 2.4. Statistics

We used independent-samples *t*-tests or Mann-Whitney-Wilcoxon tests for continuous variables and Pearson *χ* ^2^ tests or Fisher's Exact tests for categorical variables to evaluate the difference between the elderly and nonelderly. SPSS 20.0 was used for all statistical analyses. Significance was set at *P* < 0.05 (two-tailed).

## 3. Results

We enrolled 1,001 calls: 226 nontransport calls (22.6%) and 775 transport calls (77.4%). The reasons of nontransport calls were as follows: (1) the person involved refusing transport (81.1%); (2) police officer taking over the task (11.6%); (3) mission canceled on the way (0.6%); (4) misstatement (0%), and (5) others (6.7%). Of the nontransport calls with available data (72.6%, 164/226), 17.1% were made by the elderly and 82.9% by the nonelderly. The elderly with available data made only 28/304 (9.2%) nontransport calls, a significantly (*P* < 0.05) lower percentage than the 21.4% (136/635) made by the nonelderly with available data. The elderly accounted for 35.6% (276/775) of the transport calls. The estimated annual transport calls in elderly and nonelderly cases were 149 per 1000 residents and 21 per 1000 residents, respectively.

In the comparison of transport calls, the elderly had significantly more abundant histories of hypertension, diabetes, cardiovascular disease, cerebrovascular disease (CVA), cancer, end-stage renal disease (ESRD), Parkinson's disease, and Alzheimer's disease than did the nonelderly. However, they had less abundant histories of psychiatric disease and epilepsy than did the nonelderly (all *P* < 0.05) (Table 1). More elderly (46.0%) than nonelderly (36.9%) cases were transported in the morning (0:00–12:00). The elderly had significantly longer “departure-scenes” (5.8 ± 2.2 min versus 5.5 ± 2.4 min), “scene-leaves” (8.3 ± 4.3 min versus 7.3 ± 5.8 min), and total transport times (48.3 ± 13.5 min versus 46.0 ± 14.2 min) than did the nonelderly.

The elderly had significantly more nontrauma reasons (63.8% versus 34.1%) for calling EMS, such as altered mental status (16.3% versus 4.8%), dyspnea (11.6% versus 2.8%), limb weakness (7.2% versus 2.2%), abdominal pain (6.9% versus 2.4%), and cold sweating (0.8% versus 0%), than did the nonelderly; however, the elderly called less often for epilepsy and psychiatric disease than did the nonelderly (Table 2). On the contrary, the elderly called less often because of trauma (36.2% versus 65.9%) than did the nonelderly. The most common traumas for which the elderly called were traffic accidents (16.7%), slips and falls (6.2%), general trauma (5.4%), and falls (2.5%).

The elderly had significantly poorer levels of consciousness than did the nonelderly: only 70.7% of the elderly were alert (Table 3). The elderly had significantly lower Glasgow Coma Scale scores, higher systolic blood pressure, slower pulse rates, and poorer oxygen saturation than did the nonelderly during all the transport time: at the scene, on the way to the hospital, and upon arrival at the ED.

The elderly needed significantly more respiratory management, for example, nasal cannulae (13.8% versus 4.0%) and nonrebreathing oxygen masks (7.2% versus 1.6%), than did the nonelderly (Table 4). The elderly needed less trauma management than did the nonelderly. The most common types of trauma management in the elderly were irrigation (17.4%), compression because of bleeding (13.4%), long-boards (6.2%), and neck collars (4.7%). The elderly also needed more cardiopulmonary resuscitation (CPR), intravenous fluid, and 50% glucose water than did the nonelderly.

## 4. Discussion

We found that the elderly had nearly half the rate of nontransport calls and seven times annual incidence of transport calls than did the nonelderly, which suggested that the elderly had a much greater need than did the nonelderly to use the EMS. Most of the reasons for the nontransport calls were that the person involved refuses transport, which suggested that these calls were not emergent. The elderly were transported more often am and had a significantly longer transport time than did the nonelderly. The elderly had more nontrauma reasons for calling EMS, such as altered mental status, dyspnea, limb weakness, abdominal pain, and cold sweating. Most common trauma reasons for the elderly to call EMS were traffic accidents, slips and falls, general trauma, and falls. The elderly also had a poorer level of consciousness and oxygen saturation, and they needed more respiratory management and CPR during the ambulance transport.

The rate of EMS use increased exponentially with increasing age and increases in the elderly population [3, 6]. This study revealed that the elderly were responsible for 35.6% EMS transport calls in 2014, a nearly 50% increase compared with the 24% reported by Chi et al. two decades ago [7]. In another US study [3] in 2007, the elderly accounted for 38.3% of EMS transports and are expected to account for 49% in 2030. A recent study in Canada [4] reported that the elderly were responsible for 50% of EMS transport calls and 202.8 responses per 1,000 elderly cases over all. Sixty percent of the elderly ≥85 years were transported to the ED by EMS and more than 50% transported to the ED by EMS were admitted to the hospital after ED management [3, 6, 8]. Furthermore, patients ≥85 accounted for only 3% of ED visits; they accounted for more than 10% of EMS transport calls [3]. These findings suggested that the elderly have a high level of severe conditions and need transport protocols different from those used for the nonelderly.

Because their diseases and medical episodes were usually more acute and complex than those in patients in other age groups, the elderly always required more transport time and more interventions during ambulance transport. A nationwide population-based study in Taiwan reported that the highest prevalence of multiple chronic conditions was in the elderly and is still increasing (42.3% in 2000 and 64.5% in 2010, a relative increase of 52.5%) [9]. The special needs for EMS transport of the elderly were revealed in a US study [8], which reported that immobility (33%), illness (22%), requests by others (21%), instructions from healthcare providers (10%), and lack of transportation (10%) were the most common reasons for EMS transport of the elderly. EMS use increased with older age, increased deficiencies in activities of daily living, worse physical functioning, and worse social functioning [8].

The present study showed that the most common reasons for calling EMS were nontraumatic, which was compatible with studies in other countries [8, 10]. The elderly always presented vague and nonspecific signs and symptoms of illness, such as altered mental status, dyspnea, limb weakness, abdominal pain, and cold sweating. Many common diseases can exist without their typical features [11], which makes managing the elderly a more difficult task for EMS personnel.

Adapting EMS to make the service more suitable for the world's increasingly aging societies, according to current studies, is most important. Although the percentages of elderly people are different between countries, our results seem to reflect a planetary trend. Specific geriatric training and sufficient preparation of respiratory and resuscitation equipment will benefit this growing and vulnerable population. We suggest the standard operation procedure for the EMS needs to be modified as follows: (1) emergency medical dispatcher should try to take more information about the call reasons and environment of the elderly to enhance EMT management of the patients and (2) EMS should consider dispatching higher level of emergency medical technician (EMT) such as emergency medical technician-paramedics (EMT-P) with more experience and the ambulance with more resuscitation equipment for the elderly if needed. We also suggest that some government policies need to be changed for the elderly as follows: (1) redefining the drive license system to ensure that licensed elder driver has the ability to keep a safety drive to avoid traffic accident [12]; (2) construction of EMS database for the elderly who live alone to help the dispatched EMT get more and detailed information at the first timing; and (3) suggesting that the elderly live in the first floor to help EMT get the patient out in case of emergency.

This study has some limitations. First, we analyzed 1,001 EMS calls; however, the number was relatively small given our objective. Second, the data came from only one dedicated ambulance corps in a Taiwan city; thus it might not be representative of the general population of this country or any other. Additional studies with substantially larger samples and call records from multiple ambulance corps are warranted. Third, another possible explanation for the fact that the elderly had less nontransport calls than did the nonelderly is that EMT may be more cautious for the elderly and prefer to transport them to the ED. Fourth, we did not investigate the outcome of the patients, a goal of EMS, which also needs further studies to clarify this issue.

## 5. Conclusions

This study found that the elderly called EMS when they really needed it. They always called EMS for nontraumatic reasons such as altered mental status, dyspnea, limb weakness, abdominal pain, and cold sweating. Because the medical episodes for which the elderly needed to be transported to the ED were generally more acute and complex than those of the nonelderly, more time and more respiratory and resuscitation management were also required during their EMS transport. Therefore, adapting the EMS including training, operation, and government policies to an aging society in the future is mandatory.

## Figures and Tables

**Table 1:** Comparison of age, gender, medical history, and transport time between the elderly and nonelderly in the transport calls.

Variables	Elderly	Nonelderly	Total	*P*
	*n* = 276	*n* = 499	*n* = 775	
Age (years)	78.1 ± 8.1	39.6 ± 17.4	53.4 ± 23.6	<0.001
Gender				0.556
Male	151 (54.7)	262 (52.5)	413 (53.3)	
Female	125 (45.3)	237 (47.5)	362 (46.7)	
Medical history^*∗*^				
Hypertension	131 (47.5)	80 (16.0)	211 (27.2)	<0.001
Diabetes	108 (39.1)	57 (11.4)	165 (21.3)	<0.001
Cardiovascular disease	66 (23.9)	42 (8.4)	108 (13.9)	<0.001
CVA	34 (12.3)	13 (2.6)	47 (6.1)	<0.001
Cancer	29 (10.5)	18 (3.6)	47 (6.1)	<0.001
COPD/asthma	17 (6.2)	16 (3.2)	33 (4.3)	0.059
ESRD	14 (5.1)	11 (2.2)	25 (3.2)	0.038
Parkinson's disease or Alzheimer's disease	10 (3.6)	0 (0)	10 (1.3)	<0.001
Liver disease	9 (3.2)	22 (4.4)	31 (4.0)	0.3
Psychiatric disease	6 (2.2)	29 (5.8)	35 (4.5)	0.027
Epilepsy	0 (0)	12 (2.4)	12 (1.5)	0.032
Day separation of transport				0.013
a m	127 (46.0)	184 (36.9)	311 (40.1)	
pm	149 (54.0)	315 (63.1)	464 (59.9)	
Transport time (minutes)				
Departure-scene	5.8 ± 2.2	5.5 ± 2.4	5.6 ± 2.3	0.048
Scene-leave	8.3 ± 4.3	7.3 ± 5.8	7.7 ± 5.3	0.011
Leave-arrival at ED	7.8 ± 2.9	7.5 ± 2.7	7.6 ± 2.8	0.140
Arrival at ED-leave of ED	10.1 ± 6.9	10.0 ± 7.5	10.0 ± 7.3	0.879
Leave ED-return	16.4 ± 8.6	15.7 ± 7.6	15.9 ± 7.9	0.245
Total time	48.3 ± 13.5	46.0 ± 14.2	46.8 ± 14.0	0.025

Data are number (%) or mean ± standard deviation (SD). CVA, cerebrovascular disease; COPD, chronic obstructive pulmonary disease; ESRD, end-stage renal disease; ED, emergency department.

^*∗*^One patient may have multiple medical histories.

am: 00:00–12:00; pm: 12:00–24:00.

**Table 2:** Comparison of the reasons for calling EMS between the elderly and nonelderly in the transport calls.

Variables	Elderly	Nonelderly	Total	*P*
	*n* = 276	*n* = 499	*n* = 775	
Nontrauma	176 (63.8)	170 (34.1)	346 (44.7)	<0.001
Altered mental status	45 (16.3)	24 (4.8)	69 (8.9)	<0.001
Dyspnea	32 (11.6)	14 (2.8)	46 (5.9)	<0.001
Limb weakness	20 (7.2)	11 (2.2)	31 (4.0)	0.001
Abdominal pain	19 (6.9)	12 (2.4)	31 (4.0)	0.002
Headache/dizziness/syncope	19 (6.9)	24 (4.8)	43 (5.5)	0.227
Nausea/vomiting/diarrhea	12 (4.3)	15 (3.0)	27 (3.4)	0.329
Chest pain	9 (3.3)	8 (1.6)	15 (1.9)	0.131
Fever	9 (3.3)	13 (2.6)	22 (2.8)	0.653
OHCA	8 (2.9)	5 (1.0)	13 (1.7)	0.075
Cold sweating	4 (0.8)	0 (0)	4 (0.8)	0.016
Foreign-body aspiration	2 (0.7)	3 (0.6)	5 (0.6)	>0.95
Epilepsy	2 (0.7)	15 (3.0)	17 (2.2)	0.038
Fell down on the road	1 (0.4)	4 (0.8)	5 (0.6)	0.465
Drowning	1 (0.4)	0 (0)	1 (0.1)	0.178
Psychiatric disease	0 (0)	21 (4.2)	21 (2.7)	0.001
Drug poisoning	0 (0)	3 (0.6)	3 (0.4)	0.197
Suicide	0 (0)	2 (0.4)	2 (0.4)	0.541
Emergency delivery	0 (0)	2 (0.4)	2 (0.3)	0.292
CO poisoning	0 (0)	1 (0.2)	1 (0.1)	>0.95
Trauma	100 (36.2)	329 (65.9)	429 (55.4)	<0.001
Traffic accident	46 (16.7)	259 (51.9)	305 (39.4)	<0.001
Slip	17 (6.2)	14 (2.8)	31 (4.0)	0.023
General trauma^*∗*^	15 (5.4)	31 (11.2)	46 (5.9)	0.661
Fall	7 (2.5)	6 (1.2)	13 (1.7)	0.166
Penetration	2 (0.7)	0 (0)	2 (0.3)	0.057
Fighting	1 (0.4)	4 (0.8)	5 (0.6)	0.465
OHCA	1 (0.4)	0 (0)	1 (0.1)	0.178
Burn	0 (0)	3 (0.6)	3 (0.4)	0.197

Data are number (%) or mean ± standard deviation (SD). EMS, emergency medical services; CO, carbon monoxide; OHCA, out-of-hospital cardiac arrest.

^*∗*^Injury to the head, chest, abdomen, back, and limbs.

**Table 3:** Comparison of vital signs between the elderly and nonelderly in the transport calls.

Variables	Elderly	Nonelderly	Total	*P*
	*n* = 276	*n* = 499	*n* = 775	
Consciousness level				<0.001
Alert	195 (70.7)	436 (87.4)	631 (81.4)	
Verbal	37 (13.4)	34 (6.8)	71 (9.2)	
Pain	28 (10.1)	19 (3.8)	47 (6.1)	
Unresponsive	16 (5.8)	10 (2.0)	26 (3.4)	
At the scene				
GCS	13.0 ± 3.6	14.3 ± 2.2	13.8 ± 2.8	<0.001
SBP (mmHg)	140.0 ± 48.9	129.1 ± 38.3	133.0 ± 46.7	0.001
Pulse rate (1/min)	86.1 ± 26.3	93.6 ± 21.3	90.9 ± 23.4	<0.001
Respiratory rate (1/min)	18.8 ± 4.2	18.9 ± 2.8	18.9 ± 3.4	0.698
Oxygen (SpO_2_%)	91.8 ± 18.1	96.3 ± 9.7	94.7 ± 13.5	<0.001
On the way to hospital				
GCS	13.4 ± 3.1	14.3 ± 2.3	14.0 ± 2.7	<0.001
SBP (mmHg)	143.9 ± 44.5	134.1 ± 34.6	137.6 ± 38.7	0.002
Pulse rate (1/min)	86.5 ± 23.7	92.2 ± 20.1	90.2 ± 21.6	0.001
Respiratory rate (1/min)	18.9 ± 4.0	18.9 ± 2.7	18.9 ± 3.3	0.964
Oxygen (SpO_2_%)	94.1 ± 15.6	96.8 ± 9.0	95.9 ± 11.9	0.008
Arrival at ED				
GCS	13.5 ± 3.2	14.3 ± 2.2	14.0 ± 2.6	0.006
SBP (mmHg)	148.4 ± 42.0	137.1 ± 34.0	141.1 ± 37.4	<0.001
Pulse rate (1/min)	86.7 ± 25.6	91.8 ± 21.0	90.0 ± 22.8	0.006
Respiratory rate (1/min)	18.8 ± 4.1	19.0 ± 3.7	18.9 ± 3.8	0.607
Oxygen (SpO_2_%)	94.3 ± 15.6	96.8 ± 9.1	95.9 ± 11.9	0.014

Data are number (%) or mean ± standard deviation (SD). GCS, Glasgow Coma Scale; SBP, systolic blood pressure; ED, emergency department.

**Table 4:** Comparison of emergency managements between the elderly and nonelderly in the transport calls.

Variables	Elderly	Nonelderly	Total	*P*
	*n* = 276	*n* = 499	*n* = 775	
Respiratory managements				
Nasal cannula	38 (13.8)	20 (4.0)	58 (7.5)	<0.001
Nonrebreathing mask	20 (7.2)	8 (1.6)	28 (3.6)	<0.001
Simple mask	15 (5.4)	17 (3.4)	32 (4.1)	0.158
Bag-valve-mask	8 (2.9)	8 (1.6)	16 (2.1)	0.192
Laryngeal mask airway	7 (2.5)	4 (0.8)	11 (1.4)	0.059
Venturi mask	2 (0.7)	2 (0.4)	4 (0.5)	0.337
Oral airway	0 (0)	0 (0)	0 (0)	NA
Nasal airway	0 (0)	5 (1.0)	5 (0.6)	0.101
Suction	0 (0)	1 (0.2)	1 (0.1)	0.307
Heimlich maneuver	0 (0)	0 (0)	0 (0)	NA
Trauma managements				
Irrigation	48 (17.4)	188 (37.7)	236 (30.5)	<0.001
Compression of bleeding	37 (13.4)	162 (32.5)	199 (25.7)	<0.001
Long-board	17 (6.2)	40 (8.0)	57 (7.4)	0.261
Neck collar	13 (4.7)	36 (7.2)	49 (6.3)	0.161
Splint	8 (2.9)	16 (3.2)	24 (3.1)	0.394
KED	3 (1.1)	1 (0.2)	4 (0.5)	0.103
Other managements				
Elevation of head or leg	142 (51.4)	107 (21.4)	249 (32.1)	<0.001
Warming	95 (34.4)	68 (13.6)	163 (21.0)	<0.001
Monitoring vital signs	31 (11.2)	113 (22.6)	144 (18.6)	<0.001
Psychological support	19 (6.9)	62 (12.4)	81 (10.5)	0.023
Assist delivery	0 (0)	0 (0)	0 (0)	NA
Cardiopulmonary resuscitation				
EMT	8 (2.9)	4 (0.8)	12 (1.5)	0.031
AED use	7 (2.5)	6 (1.2)	13 (1.7)	0.154
Bystander	1 (0.4)	3 (0.6)	4 (0.5)	0.367
Shock	1 (0.4)	1 (0.2)	2 (0.3)	0.369
Drug managements				
Intravenous fluid	18 (6.5)	6 (1.2)	24 (3.1)	<0.001
Oral glucose solution	11 (4.0)	3 (0.6)	14 (1.8)	0.001
Nitroglycerine pill	0 (0)	0 (0)	0 (0)	NA
Bronchodilator	0 (0)	0 (0)	0 (0)	NA
Medical director online				
50% glucose water	14 (5.1)	1 (0.2)	15 (1.9)	<0.001
Albuterol (Ventolin)	1 (0.4)	2 (0.4)	3 (0.4)	0.403
Epinephrine	0 (0)	0 (0)	0 (0)	NA
Atropine	0 (0)	0 (0)	0 (0)	NA
Nitroglycerine	0 (0)	0 (0)	0 (0)	NA

Data are number (%) or mean ± standard deviation (SD). NA: not applicable; KED: Kendrick Extrication Device; EMT: emergency medical technician; AED: automatic electrical device.
